# Health Diplomacy and the Enduring Relevance of Foreign Policy Interests

**DOI:** 10.1371/journal.pmed.1000226

**Published:** 2010-04-20

**Authors:** Harley Feldbaum, Joshua Michaud

**Affiliations:** Global Health and Foreign Policy Initiative, Johns Hopkins School of Advanced International Studies, Washington, D.C., United States of America; London School of Hygiene & Tropical Medicine, United Kingdom

## Abstract

Harley Feldbaum and Joshua Michaud consider the important interplay between foreign policy and global health interests, and introduce a series on Global Health Diplomacy beginning this week in *PLoS Medicine*.

**Summary Points**

- The public health community has seized upon the concept of health diplomacy to raise the profile of health in the practice of foreign policy.
- Diverse definitions of health diplomacy represent divergent perspectives on the use and political neutrality of health interventions.
- Foreign policy priorities often determine political priority and funding for global health issues.
- The use of health interventions by states and non-state actors to achieve ulterior foreign policy objectives is a controversial but growing part of health diplomacy.
- Foreign policy interests are critical to understanding global health diplomacy.

*This article is part of the* PLoS Medicine *Global Health Diplomacy series.*

## Introduction

The rise of global health issues within the world of foreign policy is precipitating great interest in the concept and practice of health diplomacy. Much discussion of this new field, particularly within the global health community, has narrowly focused on how diplomatic negotiations and foreign policy can be used to support global health goals [1],[2]. Recent articles claim, for example, that “foreign policy is now being driven substantially by health” [3], and that health can move “foreign policy away from a debate about interests to one about global altruism” [4].

New and unprecedented opportunities to bolster global health through diplomacy have emerged, but claims that health now drives foreign policy fail to appreciate how significantly traditional foreign policy interests continue to shape health diplomacy. Foreign policy interests play a critical role in determining which global health issues achieve political priority and attract funding. In addition, an important, but less analyzed trend involves the increasing use of health interventions as *instruments* to advance foreign policy interests. Countries are increasingly using health initiatives as a means to improve security, project power and influence, improve their international image, or support other traditional foreign policy objectives.

This paper provides an introduction to the *PLoS Medicine* series on global health diplomacy. Our paper reviews recent research in the field of global health diplomacy, discussing why only select global health issues rise in political priority, examining health diplomacy initiatives driven primarily by foreign policy interests, and seeking to illuminate the constellation of interests involved in health diplomacy. The principal message is that, despite recent commentary to the contrary, foreign policy interests are of primary and enduring importance to understanding the potential and limits of health diplomacy.

## Health Diplomacy

Recent attention to health diplomacy belies widely divergent usages of the term, and a concerning lack of critical thinking on the consequences of the deeper integration of global health into foreign policy agendas [5]. The public health community has offered multiple definitions of health diplomacy [6],[7], focusing on the field being driven by globalization, diverse actors beyond nation-states, health negotiations, health impact of non-health negotiations, and most importantly the normative goal of using foreign policy to support global health. For example, Kickbusch and colleagues write that “‘global health diplomacy’ aims to capture these multi-level and multi-actor negotiation processes that shape and manage the global policy environment for health” [8]. However, other conceptions of health diplomacy deemphasize both negotiations and the primary role of global health, instead describing efforts to improve health within the larger context of supporting state interests. For example, Fauci defines health diplomacy as “winning the hearts and minds of people in poor countries by exporting medical care, expertise and personnel to help those who need it most,” [9] while a former US Secretary of Health and Human Services asks, “What better way to knock down the hatred, the barriers of ethnic and religious groups that are afraid of America, and hate America, than to offer good medical policy and good health to these countries?” [10]. In these examples, the humanitarian benefits of health interventions are justified by the objectives of foreign policy.

These different conceptions of health diplomacy represent divergent perspectives on the use and political neutrality of health interventions that will have major implications for the future of global health (Box 1). This paper, and the related papers in the *PLoS Medicine* series, will critically explore this tension between global health and foreign policy.

**Box 1. Definitions of Foreign Policy, Diplomacy, and Global Health**

**Foreign policy** is the “substance, aims and attitudes of a state's relations with others,” and may be defined as the “activity whereby state actors act, react and interact” between the “internal or domestic environment and an external or global environment” [62].

**Diplomacy** is the art and practice of conducting international relations, and “provides one instrument that international actors use to implement their foreign policy” [63].

**Global health** “places a priority on improving health and achieving equity in health for all people worldwide… emphasises transnational health issues, determinants, and solutions [and] involves many disciplines within and beyond the health sciences” [64].

## Which Global Health Issues Achieve Foreign Policy Priority?

Health issues have traditionally resided in a “low politics” position in foreign policy practice, but in recent years, certain health issues have received political attention at the highest levels of national and international politics [11],[12]. The threats from bioterrorism, infectious diseases (including HIV/AIDS, SARS, XDR-TB, avian influenza A (H5N1), and pandemic influenza A (H1N1), and an increasing awareness of the link between health and economic development [13],[14] have each played a role in linking health to the traditional foreign policy goals of protecting state security and promoting national economic interests. The perception that major disease burdens can contribute to the weakening of state capacity and the destabilization of states has connected with growing concerns about the threat of weak and failed states [15],[16]. Similarly, the need to rebuild health systems in Iraq, Afghanistan, and other conflict and post-conflict areas as a part of counterinsurgency and nation-building efforts, has further intertwined health and national security objectives in the eyes of foreign policymakers [17],[18].

**Figure 1:**
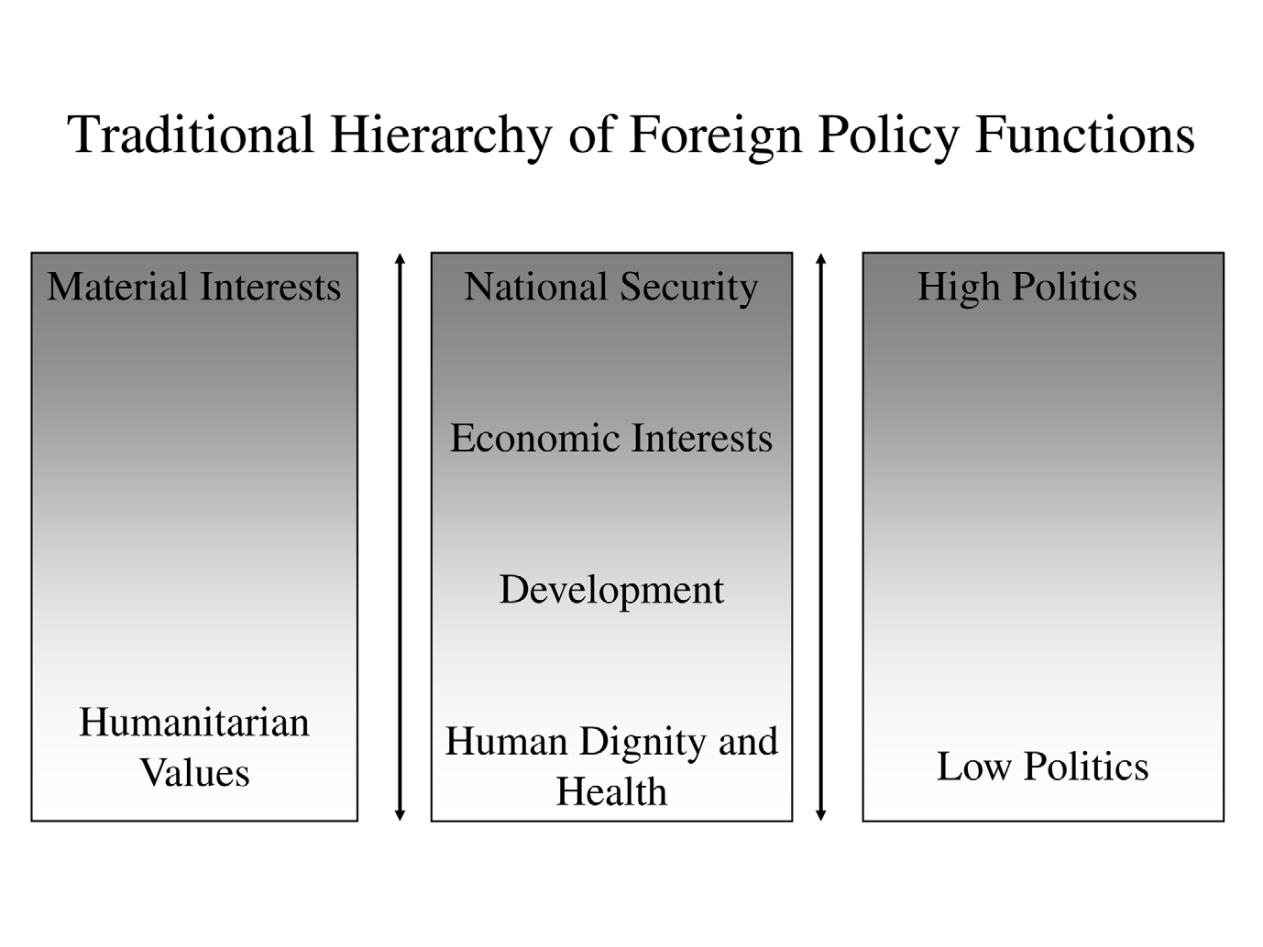
Health as “Low Politics.” Adapted from Fidler (2005) [61].

In each of these scenarios, political priority was placed on a health issue because of its perceived potential impact on one or more national security, economic, or foreign policy interests (Figure 1). The strength of the relationship between a health issue and the national interests of powerful states may be crudely measured by the amount of funding and political attention the issue receives. For instance, the many billions of dollars invested after 2001 in biodefense by wealthy countries is attributable to the perceived national security threat of a bioterrorist attack, despite bioterrorism causing only a small number of deaths to date compared to deaths from naturally occurring diseases [19].

Similarly, the threat that SARS and pandemic influenza posed to the health security and economic well-being of wealthy states pushed these diseases into the highest levels of national and international political discourse. Another example of the foreign policy–global health linkage was the timing of President George W. Bush's launch of the President's Emergency Plan for AIDS Relief (PEPFAR), which was purposely announced in the same 2003 State of the Union speech that outlined the case for invading Iraq, in order to offer a softer, humanitarian side to US foreign policy in advance of the invasion [20].

Those global health issues for which a direct link to core economic, foreign, or security interests is neither perceived nor proved will continue to be subjugated to other foreign policy priorities, regardless of the strength of the scientific evidence mustered in their favor [21]–[23]. This explains why some global health priorities, including chronic diseases, road traffic injuries, and the social determinants of health, have failed to receive attention and funding commensurate with their immense burden of disease. States simply do not perceive these issues as having significant implications for national security, economic well-being, or foreign assistance objectives.

## Health Diplomacy Driven by Foreign Policy Interests

Not only do foreign policy interests drive which global health issues garner funding and attention, but state and non-state actors alike are increasingly turning to health interventions to achieve non-health goals. Behind this trend is a growing perception that health can be an effective “soft power” tool for foreign policy (in contrast to the “hard power” of military force) [24]–[26].

For example, the US military is increasingly incorporating health (alongside other development initiatives) into their operations. These activities include the well-publicized use of the US Navy hospital ships Mercy and Comfort, as well as amphibious assault ships, to provide short-term medical care to underserved citizens around the world [27]. The US military's Joint Task Force-Horn of Africa (JTF-HOA) not only conducts operations against terrorists in the region, but also digs wells, builds schools, and provides medical care [28]. US military forces conduct Medical Civil-Assistance Programs in Iraq and Afghanistan as part of “supporting pacification, gathering local intelligence, or rewarding locals for their cooperation” [29]. Provincial Reconstruction Teams in Iraq and Afghanistan, which involve civilian and military personnel, also work on improving health conditions as part of the overall counterinsurgency strategy [30].

In short, US strategic interests in “winning hearts and minds” have incorporated health initiatives as part of that fight in a number of contexts. As one study of JTF-HOA observed, “using US military assets to perform a humanitarian mission serves a dual purpose. It shows the face of American compassion to a skeptical population while also giving the military an eye on activity in the area. Winning hearts and minds with an ear to the ground is the new American way of war” [28]. These efforts are likely to continue, despite criticism of militarized aid [31] and a lack of ability to demonstrate effectiveness [32], because most experts believe future conflicts will resemble counterinsurgencies and “armed social work” more than traditional battlefield confrontations [33]. Such thinking rose to prominence with US General Petraeus's “surge” in Iraq in 2007, and is supported institutionally within the US government by the revolutionary *Counterinsurgency Field Manual* [34], the US Government Counterinsurgency Guide [35], and the 2005 Department of Defense Directive 3000.05 which defines “stability operations,” including providing health services, as “a core US military mission” [36].

While the roots of the US military's involvement in health and development activities are complex [37], US actions are influenced by the view that “the competition uses health diplomacy” [E. Bonventre, personal communication]. For example, US hospital ship missions are partly designed to counter Cuba's long-standing health diplomacy activities, which include the deployment of thousands of health professionals around the world, support for medical education of international students, and disaster relief activities [38]. Cuba's health diplomacy activities are undertaken in large part to support its own foreign policy objectives. For example, the largest Cuban health diplomacy program operates in Venezuela, where in return for medical services Cuba gains preferential pricing for Venezuelan oil [39]. Brazil is successfully leveraging its model fight against HIV/AIDS into expanded South–South assistance and leadership, accruing “access to markets and diplomatic influence” in service of Brazil's foreign policy objectives to win a seat on the United Nations Security Council and a greater voice in the international monetary system [40]. And in a sign that health diplomacy may be an area of future state competition, China has recently launched its first hospital ship, which is expected to be utilized for both humanitarian missions and to support Chinese military actions [41]. China has also increasingly supported health programs in African countries in association with its efforts to gain access to strategic resources and markets [42].

Other examples of using health to gain political legitimacy include terrorist, militant, and insurgent organizations that provide medical services to garner support from communities in which they operate. Hezbollah, the Shi'a Islamic organization deemed a terrorist organization by several countries, has become the “the most effective welfare provider in Lebanon” through its social welfare initiatives, including health services, that generate local support for its political agenda [43]. The former Sri Lankan insurgent group, the Tamil Tigers, supported health and social services to mobilize the community to its cause [44]. Similarly, Burkle reports that Iraqi insurgents “made controlling hospitals a priority because by owning the health and social services, the control of the people soon followed, as had been the pattern in Taliban-controlled Afghanistan, as well as in Pakistan” [45]. In the eyes of state and non-state actors alike, “health diplomacy” involves health interventions used to achieve strategic foreign policy goals.

## The Enduring Relevance of Foreign Policy Interests to Health Diplomacy

This foreign policy conceptualization of health diplomacy stands in stark contrast to the idea of health diplomacy held by many global health practitioners. Some global health proponents have argued that the “political, social and economic implications of health issues” have collapsed the traditional foreign policy hierarchy of interests (see Figure 1), and that “domestic and foreign, hard and soft, or high and low—no longer apply” [8]. This perspective views improving global health as the most important goal of foreign policy in and of itself, and that health diplomacy can “shape and manage the global policy environment *for health*” [italics added] [8].

Some events seemingly bolster this view that global health has triumphed over foreign policy considerations. Examples frequently cited include the negotiation and ratification of the Framework Convention on Tobacco Control (FCTC) [3], WHO's use of “travel advisories” during the SARS epidemic [46], and the national policy coordination activities of the UK and Switzerland [8].

However, far from demonstrating a health-centric move “away from interests towards global altruism” [4], we argue that these examples actually demonstrate the enduring relevance of foreign policy interests to health diplomacy. The FCTC relied on a never-before–utilized treaty-making power of the WHO to create an agreement that aims to “reduce the growth and spread of the global tobacco epidemic” [47]. Key to the adoption of this new treaty was the evidence provided by the WHO and World Bank on the economic burden that tobacco and tobacco-related diseases place on governments [48]. Support for the treaty actually reflected economic self-interest as well as concern for health.

The WHO's use of “travel advisories” to help control the international spread of SARS, which were issued without an explicit international legal mandate and imposed economic losses on countries, was a triumph of global health interests over national sovereignty [46]. However, most nations not directly affected by the advisories supported the WHO's actions in the name of protecting their own national security and economic interests [49], which in turn helped legitimate the travel advisories against the objections of affected states [50].

Even the policy coherence activities of the UK and Switzerland, often cited as evidence that “foreign policy is now being driven substantially by health,” [3] in fact signify attempts to push global health issues to be considered *alongside* other foreign policy interests. For example, the UK's *Health is Global* strategy states that the UK will “as far as feasible, evaluate the impact of our domestic and foreign policies on global health” [51], while the *Swiss Health Foreign Policy* agreement suggests “weighing up the different foreign policy interests” [52] at stake in this area. Thus despite recent commentary, global health is not preeminent in the practice of health diplomacy and foreign policy interests remain the major driver guiding the content and processes of this field.

## Political Challenges to Global Health Cooperation

Two high-profile health diplomacy challenges highlight the tension and constant interplay between foreign policy and global health interests: the avian influenza A (H5N1) virus-sharing controversy with Indonesia, and the implementation of the International Health Regulations (IHR) 2005.

Indonesia's refusal to share samples of avian influenza A (H5N1) with the WHO Global Influenza Surveillance Network demonstrates how global health and foreign policy objectives of multiple actors can become entangled. From a foreign policy perspective, Indonesia's demand for greater transparency and control over international transfer of its virus samples is understandable because these positions support the country's material interests in trying to ensure equitable access to pharmaceuticals and medical manufacturing capacity for its own vulnerable population [53]. The previous Indonesian Health Minister's championing of this issue was also, to some extent, designed for domestic political consumption through generating the perception of standing up to powerful foreign interests [54]. Western countries, on the other hand, feared the human and economic impact of delayed detection of an emerging influenza pandemic and wish to avoid setting the precedent of acquiescing to “viral blackmail.” The global health community's reaction to these events has been split, because Indonesia's actions are seen as undermining global influenza surveillance [55], but also as a clarion call to overturn long-standing inequities in the global pharmaceutical market [56]. Both Indonesia's actions and the various global actors' responses have complex roots in self-interest, and domestic and international politics.

As for the IHR 2005, its negotiation and implementation also demonstrate the complexity of the global health and foreign policy nexus. The international agreement represents a “radically” new system of global health diplomacy, which “privilege[s] global health governance over state sovereignty,” and thus the objectives of global health over foreign policy considerations [57]. However, the IHR were adopted because they served powerful state interests, and accordingly some developing countries view the IHR as an instrument of the foreign policy and national security interests of developed countries seeking protection from epidemics emanating abroad, and therefore as only an extension of age-old power politics [58],[59]. Successfully implementing the IHR will require balancing different countries' health and foreign policy objectives, with the scientific and public health requirements for effective global disease surveillance and response. These two cases demonstrate the tensions between global health and the foreign policy objectives of different states that will complicate and define the future of global health diplomacy.

## Foreign Policy Interests and Health Diplomacy Challenges

Further consideration of the interplay between foreign policy and global health interests is the focus of a series of related articles on global health diplomacy in *PLoS Medicine*. Beginning with a case study of Brazil's role in the negotiations surrounding the FCTC in this week's issue [60], the series draws from international settings, provides critical analysis of the growing interface between foreign policy and global health, and explores how global health diplomacy mediates between these two realms. Additional case studies examine whether SARS was a watershed for China's engagement in global health diplomacy and whether the controversies surrounding avian influenza A (H5N1) and pandemic influenza A (H1N1) may limit equitable access to influenza vaccine. The *PLoS Medicine* series will conclude with commentary from high-level diplomats involved in global health diplomacy, providing critical insights into current diplomatic challenges in global health.

## Abbreviations

FCTC: Framework Convention on Tobacco Control
IHR: International Health Regulations
WHO: World Health Organization
